# A painless way to customize Circos plot: From data preparation to visualization using TBtools

**DOI:** 10.1002/imt2.35

**Published:** 2022-07-04

**Authors:** Chengjie Chen, Ya Wu, Rui Xia

**Affiliations:** ^1^ State Key Laboratory for Conservation and Utilization of Subtropical Agro‐Bioresources South China Agricultural University Guangzhou China; ^2^ Guangdong Laboratory for Lingnan Modern Agriculture South China Agricultural University Guangzhou China; ^3^ School of Geography and Resources Guizhou Education University Guiyang China

**Keywords:** Circos, data visualization, genome scale, TBtools

## Abstract

Circos plots enable scientists to easily inspect big biological data genome‐widely on a macroscopic scale, but cumbersome preparation of input data and complex parameter configuration limits its application. We have developed the “Advanced Circos” function in TBtools, to provide a simple way to construct Circos plots. As an out‐of‐the‐box combo toolkit, TBtools has integrated a set of functions convenient for input data preparation. The “Advanced Circos” function is supplied with a user‐friendly interface for the customization of parameter settings and can be deployed to visualize all kinds of genomic data, such as genomic associations, alignment data, gene density, and QTL locations. In the present article, we introduce the main features of “Advance Circos” and the protocols of upstream data preparation, aiming to endow more users with the ability to use Circos plots in big genomic data exploration.

## Highlights

- An out‐of‐the‐box solution for genome‐scale data visualization
- A step‐by‐step protocol for Circos plot configuration
- Reproducible and resumable project management

## Graphical abstract

Coming with a handy toolkit, Advanced Circos in TBtools provides a painless way to construct Circos Plots starting from input file preparation. Tracks including associated regions, dot plots, line plots, bar plots, heatmap, feature tags, and so forth are all supported. Try it! Coordination transformation and graphics element manipulation are quite easy.

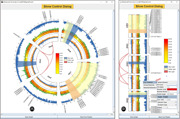

## MOTIVATION

With the rapid development of sequencing technologies and the improvement of data analysis techniques, more and more genome sequences of living organisms have been decoded. Concomitantly biology research is advanced into the postgenomic era, which requires more often an exploration of large biological data at a whole‐genome scale. In 2009, Krzywinski presented Circos, a powerful method for the visualization of big genomic data [1]. Since then, Circos has been used in numerous analyses of comparative genomics, but its utility has not been fully unlocked largely due to its complexity in plotting configurations and procedures. Although several tools were developed for quick deployment of Circos plots [1–7], there are still areas for improvement: (1) dependence on a high level of computer skills for tool installation and working under a command‐line environment, for example, Perl‐Circos; (2) preparation of input data files using extra tools; (3) limited ability of interactive edition, replotting, and collaborative sharing of intermediate visualization files of projects.

Hence, we develop the “Advanced Circos” function in TBtools [8], aiming to provide the easiest and most convenient way to create Circos plots. What users need to do is to organize tab‐delimited input files by following simple prompts in the graphic interface in TBtools. All plotting parameters can be adjusted interactively and Circos graphs can be generated and refreshed instantly. Working projects can be saved for further modification, reproduction, and sharing. In addition, TBtools, as a multifunctional toolkit, comes with a series of functions for text processing and data arrangement to assist users with an easy and quick input data preparation, providing a one‐stop solution for Circos plot creation.

## DATA CATEGORIES

**Figure 1:**
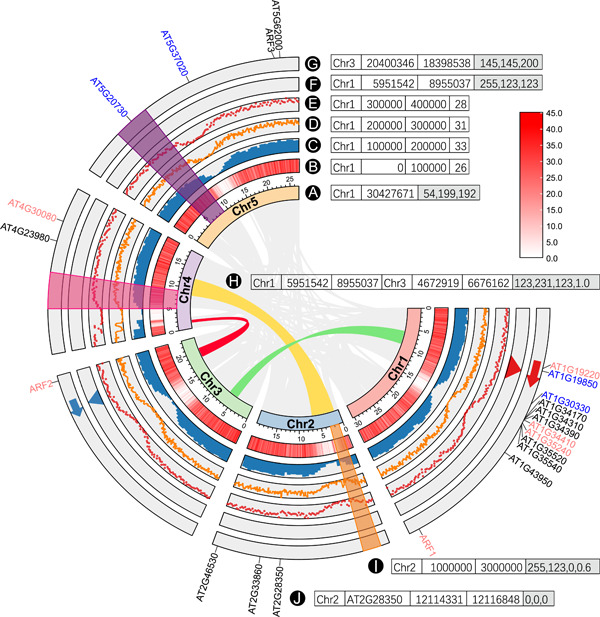
Data categories are shown in a demo Circos plot created by the “Advanced Circos” of TBtools. (A) Chromosome skeleton; (B) heatmap; (C) bar plot; (D) line plot; (E) point plot; (F) triangle; (G) arrow; (H) bézier curve; (I) tile; (J) text label. Adjacent to each label character is an example of their corresponding input data format. Text with gray backgrounds denotes that the corresponding column is optional.

The “Advanced Circos” function in TBtools supports the visualization of multiple tracks with either continuous or discrete genomic data. In general, these data tracks can be divided into four main categories as exemplified in Figure 1.

The first category is the chromosome skeleton (Figure 1A), presenting specific chromosomes or other genomic sequences (like scaffolds or contigs). It is the backbone of a Circos plot and is mandatory input data. The default input data is tab‐separated with two mandatory columns, one is Chromosome ID and the other Chromosome Length Information. Alternatively, the third column of an optional RGB Code can be included to specify the color of the chromosome skeleton.

The second category is tag data of specific chromosome regions (Figure 1J), which can be used to label specific intervals, such as gene or QTL (quantitative trait loci) locations. The corresponding input is a tab‐delimited file containing four mandatory columns and one optional column: Chromosome ID, Region Tag Label, Start Coordinate, End Coordinate and RGB Code (optional).

The third category shows the information on chromosome region associations (Figure 1H), commonly used to display homologous regions or chromosome interactions, and so on. This type of data is normally placed in the innermost of Circos plots. The input file is tab‐delimited with six mandatory columns and one optional column: chromosome ID, start coordinate, end coordinate, chromosome ID, start coordinate, end coordinate, and RGB Code (optional).

The fourth category is data of chromosome region statistics (Figure 1B‐G,I), which can be displayed in various ways, including continuous data (shown in heatmap, bar plot, line plot, or point plot) and discrete data (shown with triangles, arrows or tiles/rectangles). For continuous data, the input file is formatted as “chromosome ID, start coordinate, end coordinate, and a value shown in numbers” (Figure 1B–E); For discrete data, the input file format is “chromosome ID, start coordinate, end coordinate, and an RGB Code” (Figure 1F,G,I). For the arrow symbol (Figure 1G), when the start coordinate is larger than the end coordinate, it is reversed in direction. By adjusting plot spans, different tracks on the Circos plot can be stacked to achieve various combinations, as in Figures 1I and 6. The rectangle/tile track is employed to highlight chromosome intervals.

## GRAPHICS INTERFACES

### Main interface

**Figure 2:**
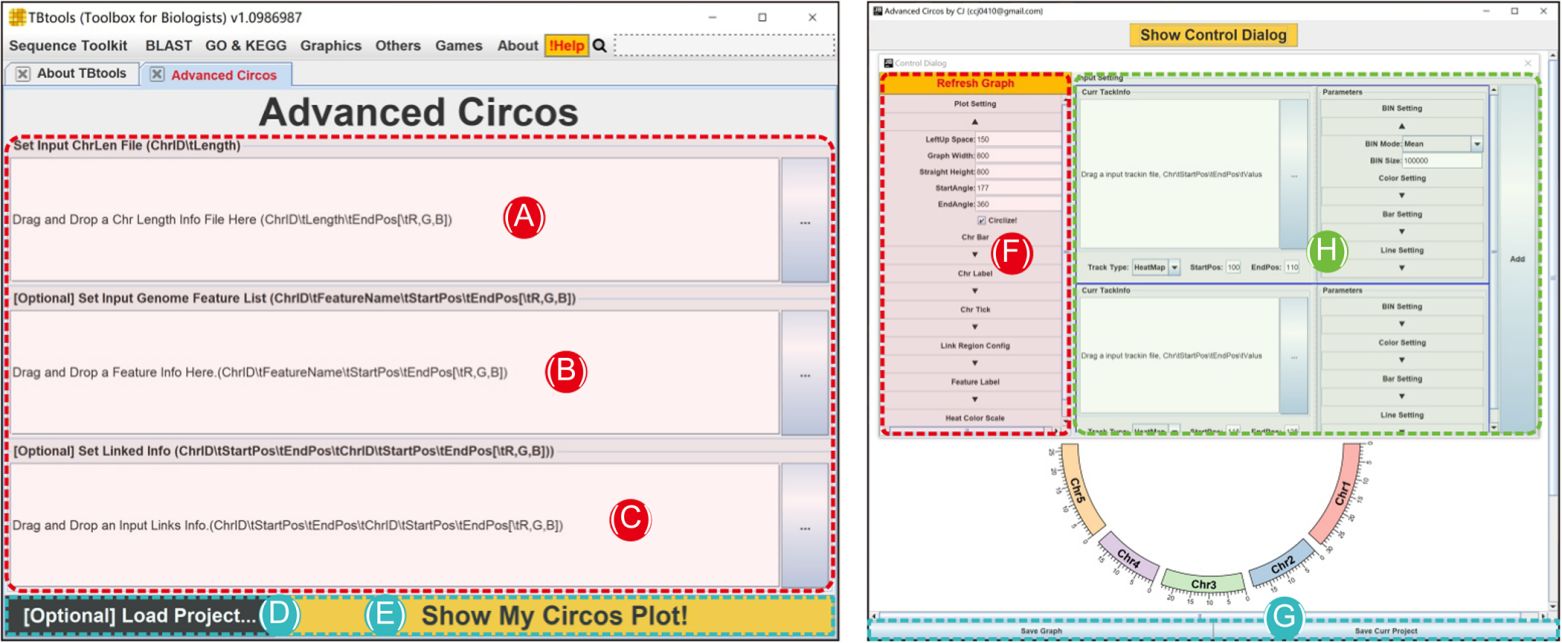
Main interface and parameter panel of advanced circos. (A–C) Input file panel; (D) button for pre‐existing project restoration; (E) button to generate genome skeleton plot; (F) global parameter settings for the Cirocs plot; (G) buttons to export plots or save project; (H) parameter settings for each chromosome region statistics track.

Open TBtools ‐> “Graphics” ‐> “Advanced Circos”, in the pop‐up interface (Figure 2A–E), there are three fields for input files and two functional buttons: 
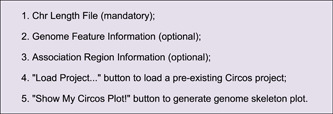


### Advanced parameter settings

Advanced Circos control panel can be revoked by clicking the “Show Control Dialog” button. The parameter setting panel can be divided into three main parts (Figure 2F,G).

1. **Save Buttons** at the bottom (Figure 2G). The “Save Graph” button is for the export of the current plot, with both bitmap and vector formats supported; the “Save Curr. Project” button can be used to save the ongoing project, which can be restored via the aforementioned “Load Project…” button. Projects can be shared directly with other users or reproduced directly on other devices;
2. **Global Parameter Settings** on the left (Figure 2F). These settings are used to control overall plotting details which are grouped into several parts: Plot Setting, Chr Bar, Chr Label, Chr Tick, Linked Region, Feature Label, Heat Color Scale. The “Refresh Graph” button on the top is used to apply adjusted parameters and refresh the ongoing plot.
3. **Track Control Parameters** on the right (Figure 2H). They are used to control visualization details of chromosome region statistics, which are also categorized into several parts, including BIN Setting, Color Setting, Bar Setting, and Line Setting. By clicking the “Add” button, a new track will be generated. For detailed effects of each parameter, a self‐test is recommended with demo data (Supporting Information 1).

## CASE DEMONSTRATION

Advanced Circos is one of the built‐in functions of TBtools, which is integrated with a collection of functions that can be used for input data preparation. In this section, we present a demonstration of how to use the Advanced Circos function of TBtools step‐by‐step to generate a Circos plot from common big biological data.

### (A) Chromosome Skeleton Preparation

Chromosome skeletons are the backbone of Circos plots. This information about a genome could be obtained from a genome sequence file using the “Fasta Stat” function in TBtools (Supporting Information: Figure S1).
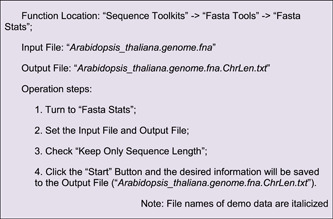


The output file (“*Arabidopsis_thaliana. genome.fna.ChrLen.txt*”) contains sequence length information of each chromosome. Users could edit it manually. For instance, we deleted the length information of two plasmid chromosomes here.
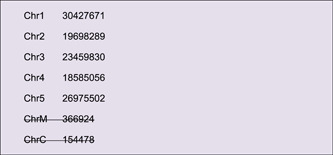


**Figure 3:**
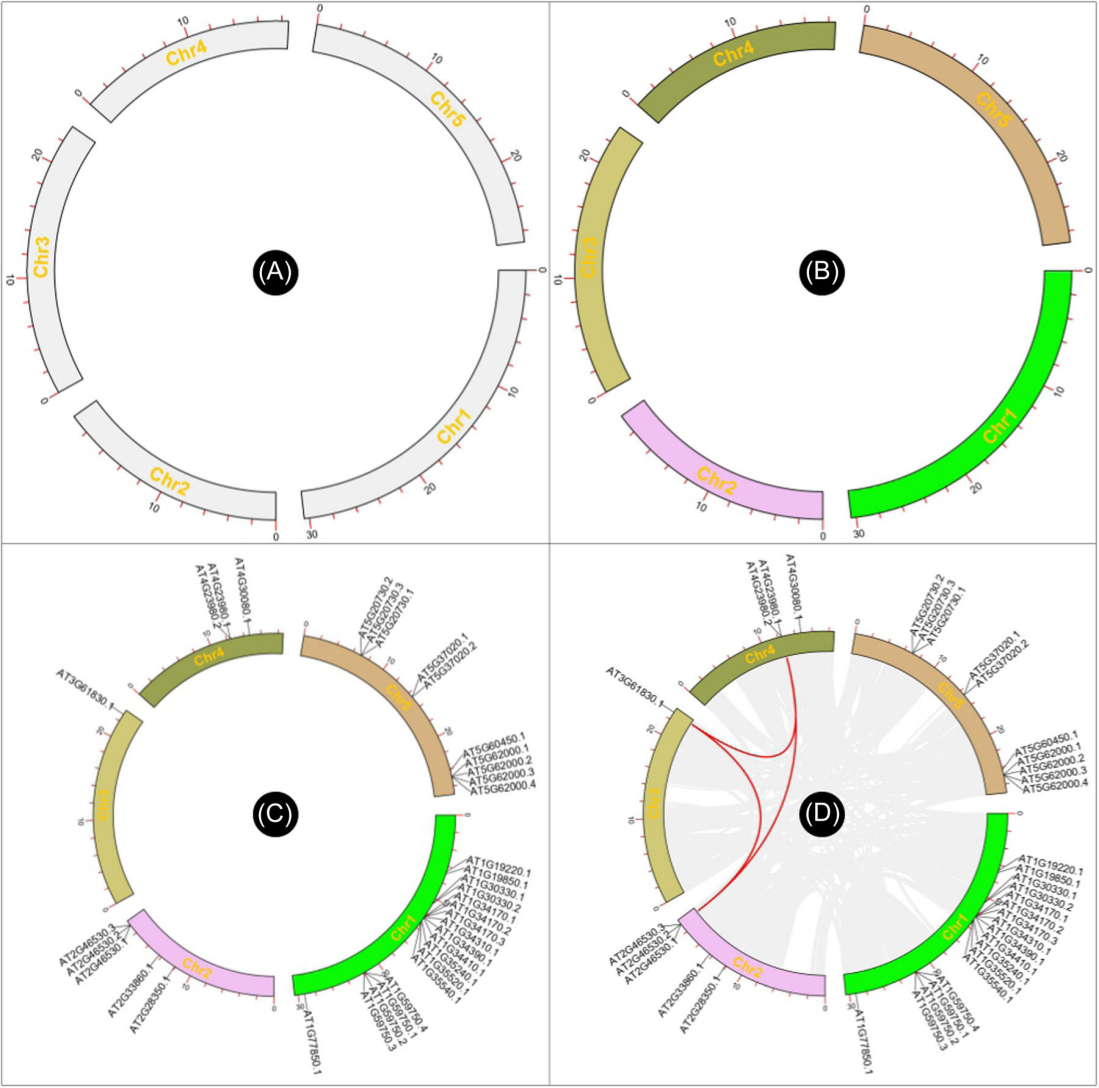
Chromosome skeleton, region tag, and region association data track of advanced Circos. (A) Basic chromosome skeleton; (B) chromosome skeleton coded with different colors; (C) gene position visualization on chromosome skeleton; (D) chromosome region association among chromosomes.

Simply drag and place the resultant file of chromosome length information into the first field in Advanced Circos (Figure 2A), press the “Show My Circos Plot!” button, and a circular plot of chromosome skeleton will be generated instantly (Figure 3A).

Different colors can be rendered to chromosomes, by adding RGB color codes to each line of chromosome length information. Users can also employ the “Discrete Color Scheme Generator” to generate a series of colors automatically (Supporting Information: Figure S2).
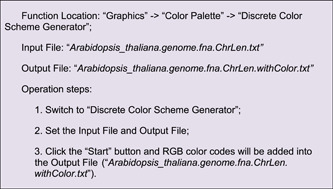


Content in the Output File will look as follows:
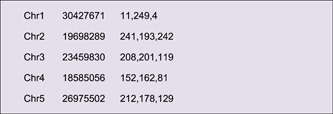


Replace the chromosome skeleton information with this file in Advanced Circos, and a colored skeleton plot will be generated (Figure 3B).

### (B) Genomic feature addition

In many cases, we would like to label positions of certain genomic features on chromosomes, such as genes, QTLs and TADs (topologically associating domains). For example, to highlight some genes, users need to get the position information of each gene first. The “GXF Gene Position & Info. extract” function of TBtools can be used to obtain corresponding genomic regions of specific genes (Supporting Information: Figure S3).
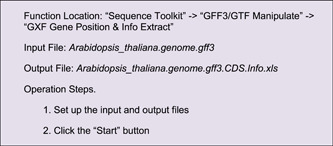


The Output File contains location information for all CDSs of Arabidopsis. Users can select the information of interest genes from this table using Excel or other text editing software. Here, we singled out genes of the ARF family in Arabidopsis (ID list of *ARF* genes had to be prepared in advance), using the “Table Row Manipulate” function in TBtools.
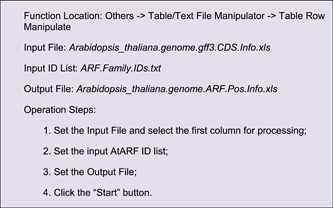


A simple edition would be required for the resultant file, i.e. keeping only the first four columns, and the location information will look as follows:
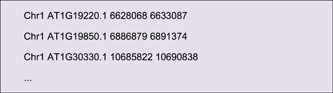


This file can be used directly for Advanced Circos visualization. Users can also append RGB codes for each gene to render different text colors. In case of feature labels overlapped in the plot, adjust the “Overlap Weight” value under “Feature Label” in the “Global Parameter Settings” to “−4” or a larger number to optimize the text interspace (Figure 3C).

### (C) Illustration of genomic region associations

Genome sequences are intra‐ or inter‐associated, such as sequence homology, chromosome interactions, and regulatory connections. Circos plots are often deployed to show genomic features of whole‐genome duplications, large segment translocations, and tandem repeats. Users can prepare relevant input files simply by using a few functions in TBtools, for instance, the “One Step MCScanX Wrapper” function [9] (Supporting Information: Figure S3).
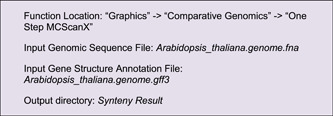


In the output directory, a file suffixed with “*. geneLinkedRegion. tab. xls” will be generated. It can be used for Advanced Circos visualization. The last column (the 8th column) of this file contains information on homologous gene pairs, which will be ignored in the Advanced Circos function. Nonetheless, users can select out association intervals of interest, adjust RGB codes, and move these lines to the head of the input file to highlight these regions. An example file will look like the following. And the resultant plot would look similar to Figure 3D. 
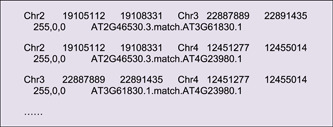


### (D) Visualization of genomic data

#### GC‐content/GCskew/N‐ratio

Nucleic acid composition is a fundamental feature of a genome. For instance, GC content is correlated with the density of coding genes and functional DNA elements; GC skew, an index measuring strand‐specific guanine and cytosine overrepresentation, can facilitate the detection of DNA replication initiation sites in bacterial circular chromosomes. Unknown base (N) denotes genome assembly quality. The function of “Fasta Window Stat” in TBtools can be employed to quickly tabulate GC content, GC skew, and N‐ratio from genome sequence files.
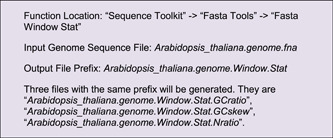


**Figure 4:**
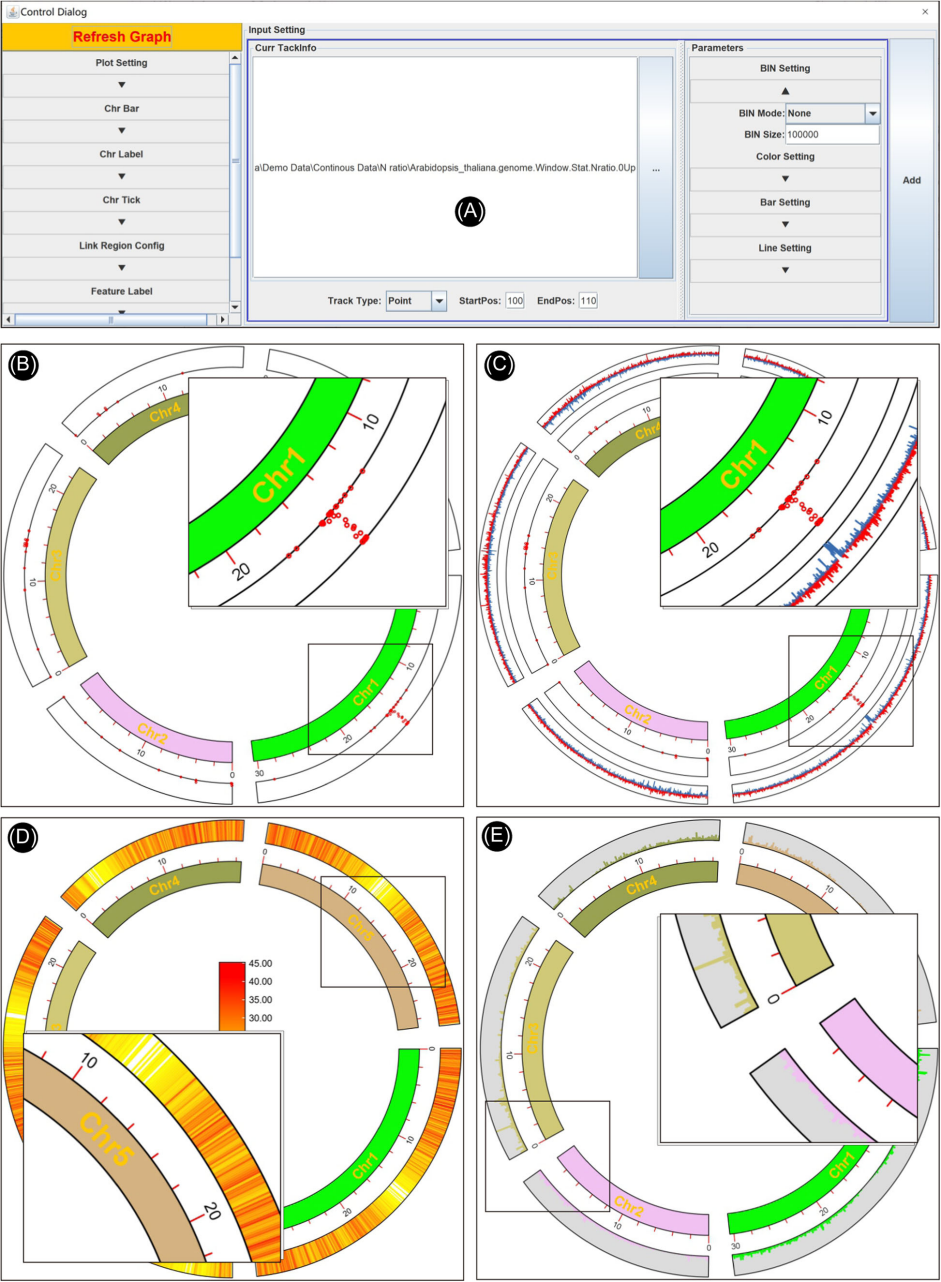
Continuous data viewed with different track types. (A) Parameter panel for a track; (B) N‐ratio profile viewed in Point plot; (C) GC skew in line plot; (D) gene density in heatmap; (E) sequencing coverage in bar plot.

These three output files are all good for Advanced Circos visualization. Here, we use the file of N‐ratio statistics as the first example (Figure 4A).
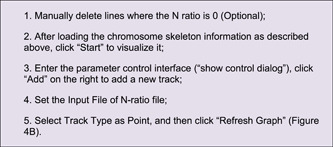


Notice that we have already used a sliding window method for the calculation of the N‐ratio, so “BIN Mode” can be set to “None.”

Similarly, we can use Line to visualize GC skew. The calculation of GC skew is bounded by 0, so we set “Sep Line Value” to 0 in Line Setting on the right to achieve different coloring for positive and negative skew values (Figure 4C).

#### Gene density

Genome‐wide gene density distribution is often viewed by Circos plots as well. TBtools has a convenient function ‐ “Gene Density Profile,” which allows users to profile gene density from gene structure annotation files, commonly in GFF3 and GTF format.
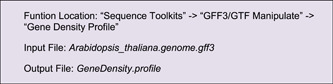


In the “Control Dialog” panel, click “Add” to get an additional Track, drag and drop the gene density information file (“*GeneDensity. profile*”), select Heatmap mode, and then refresh the plot (Figure 4D). If gene density profiles are obviously biased, users can try different color scaling modes in the “Heat Color Scale” menu at the bottom left panel for a better view. A legend of color scheme will be automatically for each heatmap track, and it can be moved around easily.

#### Coverage of sequence alignment

In many cases, besides the features of genomic sequences themselves, we are also interested to view the distribution of real NGS data over a genome, for example, the coverage of deep sequencing data. The “SAM/BAM/CRAM BIN Cov” function in TBtools can be used to prepare the input file from original alignment files generated by mapping tools (often in SAM, BAM, or CRAM formats)
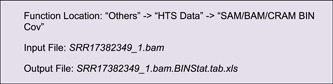


Here, we chose a bar plot to visualize alignment data. Check “Color by Chr” to make the bar coloring consistent with the colors of chromosomes. The “Bar Fill” option controls the background color of the track, which is set to gray here (Figure 4E).

#### Data of genomic variation

Similar to alignment files (for sequence coverage), in TBtools, users are also capable of processing data of genomic variation, for instance, VCF files which contain genomic information of sequence variations. The “VCF BIN Cov” function can be used for input preparation.
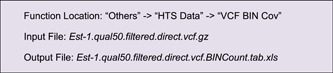


Using the heatmap mode, the hotspot regions of sequence variation on chromosomes can be viewed comprehensively. The overall pattern is largely complementary to the aforementioned gene density distribution.

#### QTLs/Arrow/TAD

All the demo data used above are continuous genomic data, “Advanced Circos” also offers various modes for displaying discrete data. We can organize QTL information according to the format requirements of the aforementioned tile track, for example:
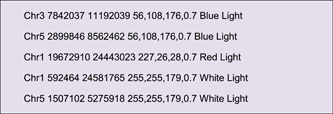


Use this data as input and select “Tile” mode to display it (Figure 5A). Notice that the last column is optional and its existence will automatically trigger legend generation. All legends can be moved easily. Besides, “Advanced Circos” can also be used to visualize TAD (with “Triangle” mode) or intervals with orientation (with “Arrow” mode) (Figure 1).

### (E) Other customization options

**Figure 5:**
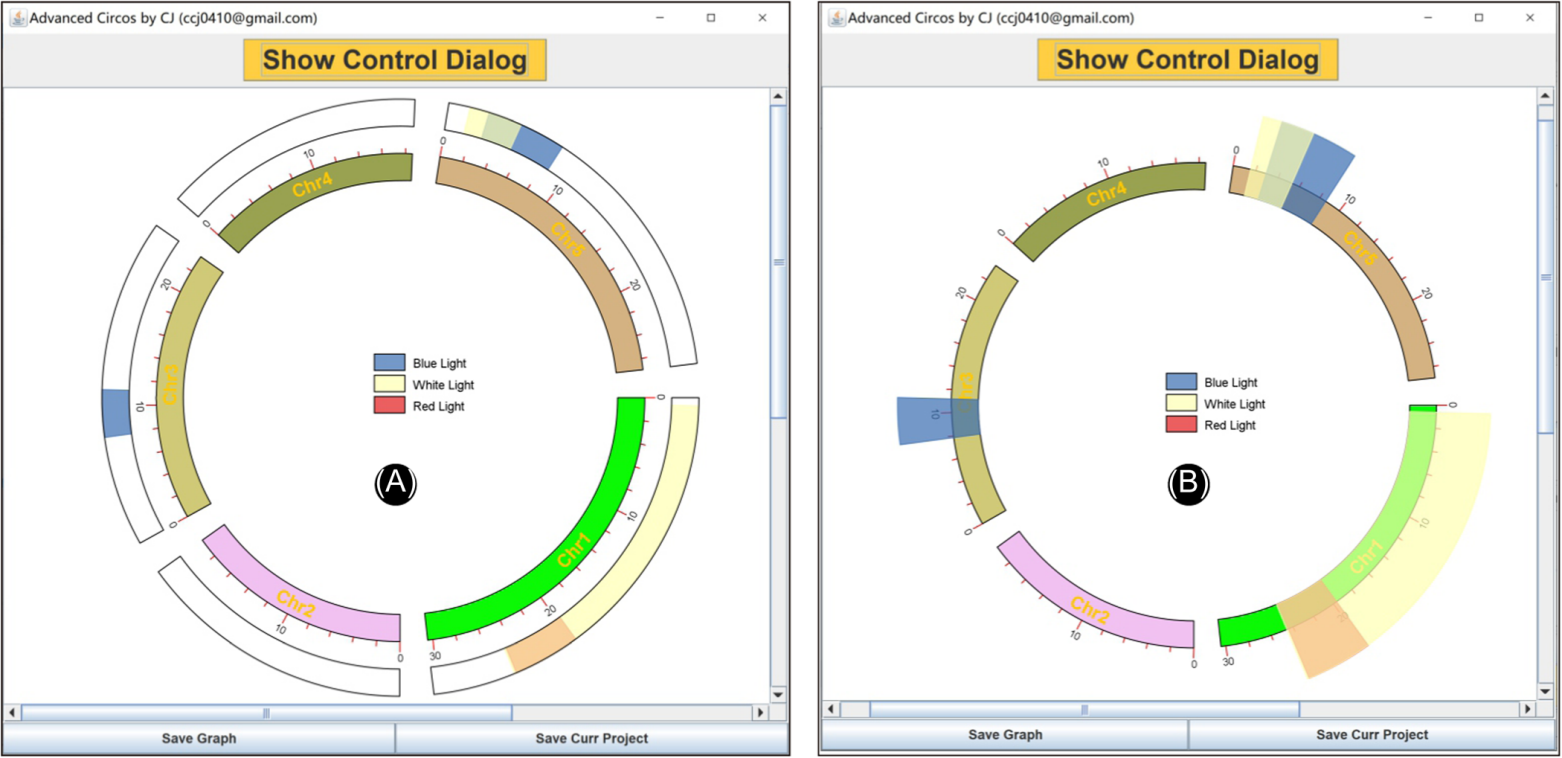
Discrete data represented with different track types. (A) Tile plot of QTLs; (B) modification of tile plot to highlight genomic intervals of interest.

#### Region highlight

The Highlight Region feature can be achieved by adjusting plotting spans (“Start Pos” and “End Pos” of the “Tile” track) of a Tile Track. Starting from Figure 5A, set “Start Pos” to 90 and “Bar Border” to “null” and refresh the image, we will get a plot like Figure 5B.

#### Track overlap

**Figure 6:**
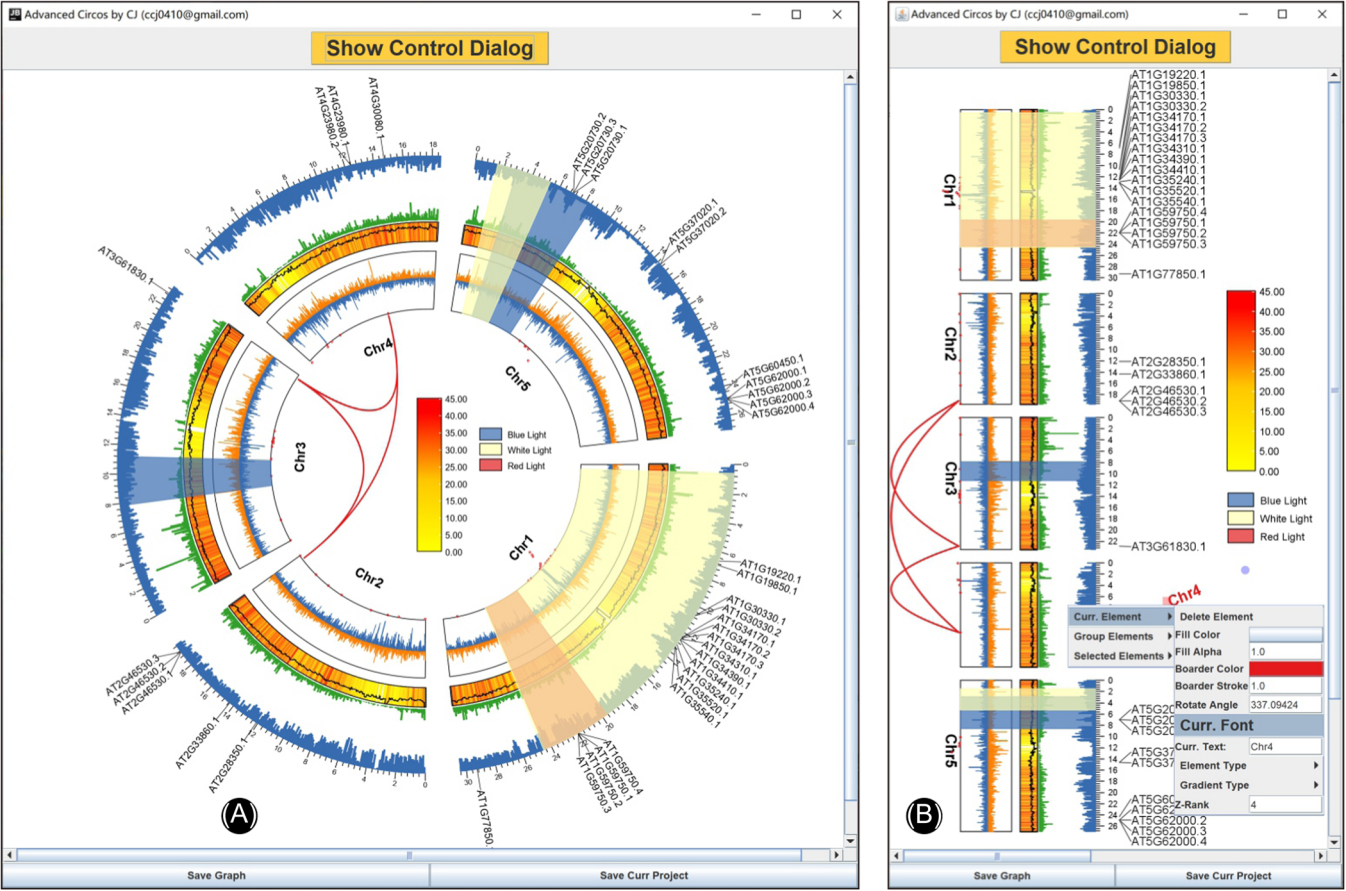
Overlapped tracks in advance Circos. (A) From the inner to outer of the Circos plot: Bézier curves for associated genomic regions; point plot for N‐ratio distribution; line plot for GCskew; heatmap for gene density profile overlapped with Line plot for GC ratio variation; bar plot for sequencing coverage; upside‐down bar plot for variant distribution; tag labels for a gene family; (B) A “straight” view of the former Circos plot.

Following the same logic of the Region Highlight feature, different tracks can be merged into one, which is also achieved by adjusting the “Start Pos” and “End Pos” of each track. By simply merging the above tracks, we could get a graph like Figure 6A.

#### Straight or circulized

Advanced Circos is developed based on the powerful interactive plotting engine “JIGplot” of TBtools we have been developing. Hence, the “Advanced Circos function” is born with the ability of switching coordinates (between Cartesian and Polar coordinates) and interactive editions. By de‐selecting the “Circulized” checkbox, we will obtain a “straight” mode of the former Circos plot (Figure 6B). In addition, users could easily edit every element in the plot, for instance, rotating elements and changing text font and color.

#### Saving, sharing, and reloading project

From the main plotting panel, click “Save Curr. Project” to save the current plot data and status to a specified directory. Users can resume work by revoking the saved project at any time directly from the Advanced Circos main interface. In addition, users can share their Circos projects by simply packaging the directory and sending it to a colleague or working from another device.

## CONCLUSION

Cumbersome data preparation, complex configuration, and extensive text collation are restraining the usage of Circos plots in scientific research. In this study, we briefly introduce plotting features and parameter interfaces of the “Advanced Circos” function in TBtools and show in detail how to use “Advanced Circos” to make information‐rich Circos plots starting from common NGS biological data. Almost all steps can be easily achieved in TBtools with simple point‐and‐click. We anticipate that the “Advanced Circos” of TBtools with this article will enable more researchers to enjoy the advantages of Circos plots to explore big biological data.

## AUTHOR CONTRIBUTIONS

Chengjie Chen and Rui Xia conceived the project; Chengjie Chen and Rui Xia designed the functions of the toolkit; Chengjie Chen performed all the Java coding. Ya Wu tested the functions and helped with the preparation of the tutorial manual and prepared the figures. Chengjie Chen and Rui Xia design the figures and wrote the manuscript. All authors read and approved the final manuscript.

## CONFLICT OF INTEREST

The authors declare no conflict of interest.

## Supporting information

Supporting information.

## Data Availability

All demo data and the corresponding version of TBtools are available at https://tbtools.cowtransfer.com/s/c60a5cfec3274f. Supplementary materials (figures, tables, scripts, graphical abstract, slides, videos, Chinese translated version, and updated materials) may be found in the online DOI or iMeta Science http://www.imeta.science/.
