# Supplementary material for: A painless way to customize Circos plot: From data preparation to visualization using TBtools
Source: Imeta. 2022 Jul 4;1(3):e35. doi: 10.1002/imt2.35 (PMC10989971; doi:10.1002/imt2.35)
Supplement: Supplementary file 1 — Supporting information. [file IMT2-1-e35-s001.docx]

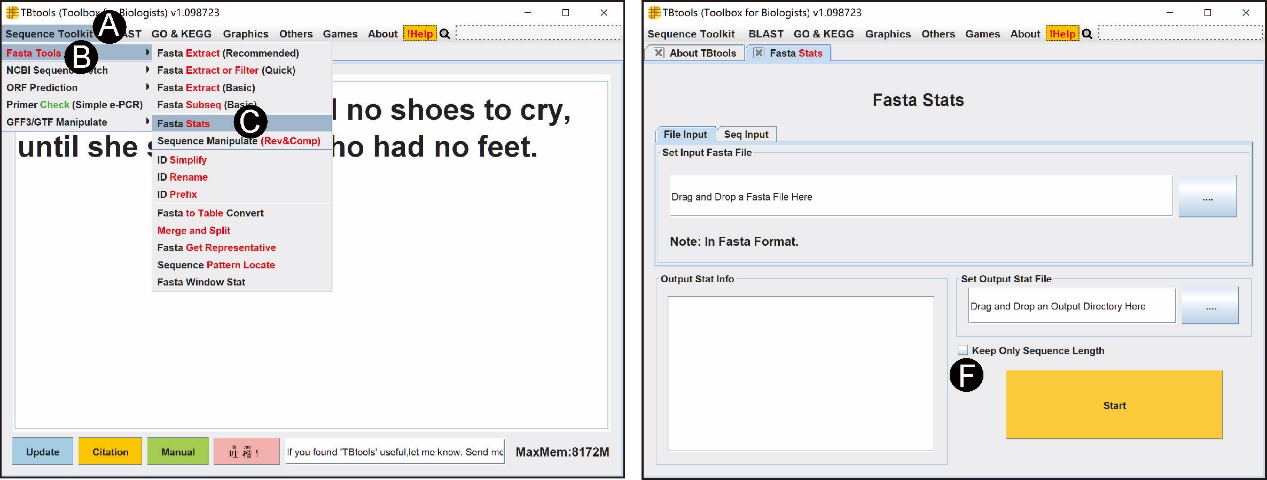


**Figure S1.** Guidance to Use "Fasta Stat" Function in TBtools. (A) Switch to the "Fasta Stat" Function; (B) The graphics user interface of the "Fasta Stat" function.


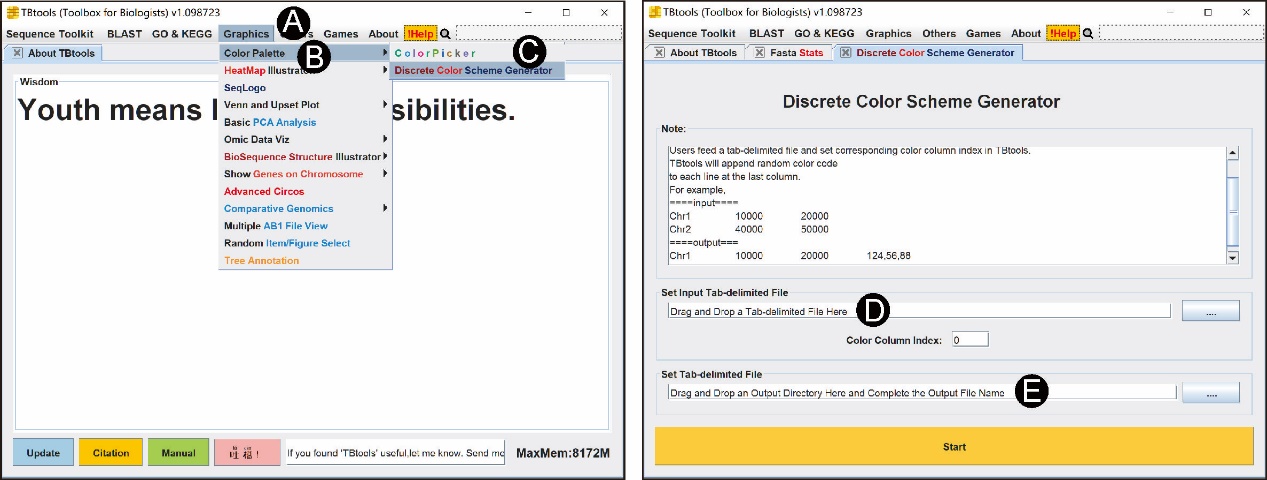


**Figure S2.** Guidance to Use "Discrete Color Scheme Generator" Function in TBtools. (A) Switch to the "Discrete Color Scheme Generator" function; (B) The graphics user interface of the "Discrete Color Scheme Generator" function.


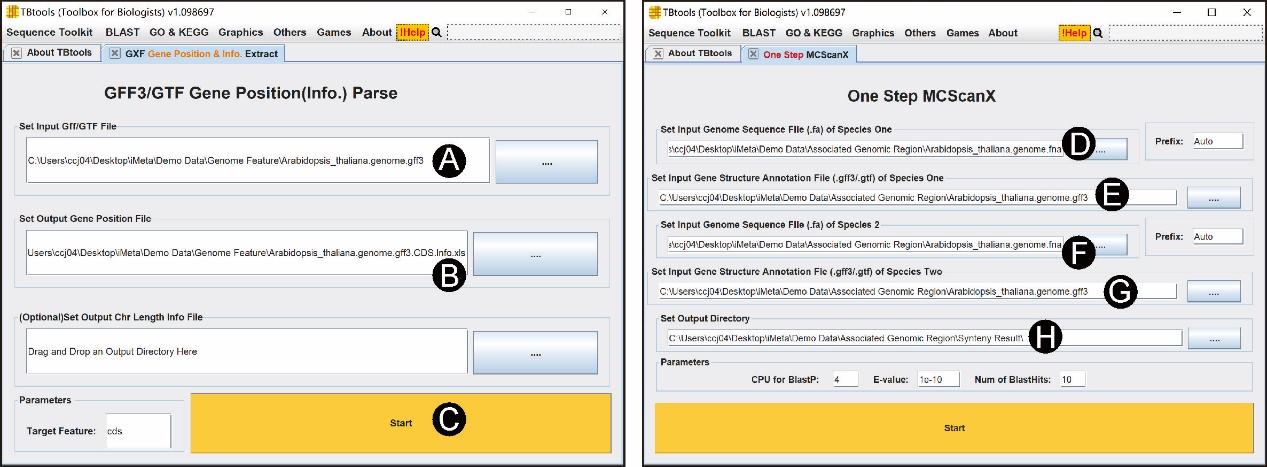


**Figure S3.** Guidance to Perform Synteny Analyses in TBtools. (A) Use the “GFF3/GTF Gene Position (info.) Parse” to extract gene positions; (B) Use the “One Step MCScanX” function to perform synteny in one click.
